# Urban Air Quality Management at Low Cost Using Micro Air Sensors: A Case Study from Accra, Ghana

**DOI:** 10.1021/acsestair.4c00172

**Published:** 2024-11-06

**Authors:** Collins Gameli Hodoli, Iq Mead, Frederic Coulon, Cesunica E. Ivey, Victoria Owusu Tawiah, Garima Raheja, James Nimo, Allison Hughes, Achim Haug, Anika Krause, Selina Amoah, Maxwell Sunu, John K. Nyante, Esi Nerquaye Tetteh, Véronique Riffault, Carl Malings

**Affiliations:** † School of Environmental, Civil, Agricultural and Mechanical Engineering, College of Engineering, University of Georgia, Athens, Georgia 30602-6113, United States; ‡ School of Sustainable Development, University of Environment and Sustainable Development, PMB, Somanya, Eastern Region, Ghana; § Clean Air One Atmosphere, Accra, Ghana; ∥ MRC Centre for Environment and Health, Environmental Research Group, Imperial College London, London W12 0BZ, U.K; ⊥ Cranfield University, School of Water, Energy and Environment, Cranfield, MK43 0AL, U.K.; # Civil and Environmental Engineering, University of California, Berkeley, Berkeley, California 94720-1234 United States; ∇ Kwame Nkrumah University of Science and Technology, KNUST, Kumasi, Ghana; ○ Lamont Doherty Earth Observatory, Columbia University, Palisades, New York 10027, United States; ● Department of Physics, University of Ghana, Legon, Accra, Ghana; □ AirGradient Ltd, Chiang Mai 50180, Thailand; ■ Environmental Protection Agency, Accra, Ghana; △ IMT Nord Europe, Institut Mines-Télécom, Univ. Lille, Centre for Energy and Environment, 59000 Lille, France; ▲ Morgan State University, Baltimore, Maryland 21251, USA & NASA Global Modelling and Assimilation Office, Goddard Space Flight Center, Greenbelt, Maryland 20771, United States

**Keywords:** High-resolution data, Ghana, Africa, Micro air sensors, PM_2.5_, Source apportionment

## Abstract

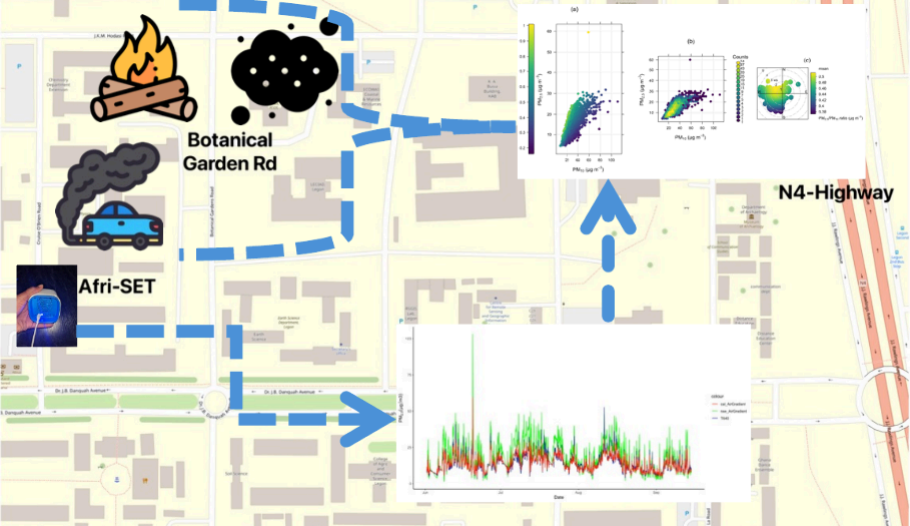

Urban air quality management is dependent on the availability of local air pollution data. In many major urban centers of Africa, there is limited to nonexistent information on air quality. This is gradually changing in part due to the increasing use of micro air sensors, which have the potential to enable the generation of ground-based air quality data at fine scales for understanding local emission trends. Regional literature on the application of high-resolution data for emission source identification in this region is limited. In this study a micro air sensor was colocated at the Physics Department, University of Ghana, with a reference grade instrument to evaluate its performance for estimating PM_2.5_ pollution accurately at fine scales and the value of these data in identification of local sources and their behavior over time. For this study, 15 weeks of data at hourly resolution with approximately 2500 data pairs were generated and analyzed (June 1, 2023, to September 15, 2023). For this time period a coefficient of determination (*r*^2^) of 0.83 was generated with a mean absolute error (MAE) of 5.44 μg m^–3^ between the pre local calibration micro air sensor (i.e., out of the box) and the reference-grade instrument. Following currently accepted best practice methods (see, e.g., PAS4023) a domain specific (i.e., local) calibration factor was generated using a multilinear regression model, and when this factor is applied to the micro air sensor data, a reduction, i.e. improvement, in MAE to 1.43 μg m^–3^ was found. Daily variation was calculated, a receptor model was applied, and time series plots as a function of wind direction were generated, including PM_2.5_/PM_10_ ratio scatter and count plots, to explore the utility of this observational approach for local source identification. The 3 data sets were compared (out of the box, domain calibrated, and reference-grade) and it was found that although there were variations in the data reported, source areas highlighted based on these data were similar, with input from local sources such as traffic emissions and biomass burning. As the temporal resolution of observational data associated with these micro air sensors is higher than for reference grade instruments (primarily due to costs and logistics limitations), they have the potential to provide insight into the complex, often hyperlocalized sources associated with urban areas, such as those found in major African cities.

## Abstract 2

Research on micro air sensor data for emission source identification is patchy. This study reports PM_2.5_ emission sources in an urban setting using relative and calibrated micro air sensor data compared to reference grade data with implications for low-cost air pollution management and control.

## 1 Introduction

Air pollution is a significant and pressing issue worldwide, with severe consequences for human health and the environment.^1−3^ It is especially problematic in Africa, where it has resulted in over 1.1 million annual premature deaths, according to recent reports.^1,4,5^ This highlights the urgent need for effective air quality monitoring and mitigation measures to protect people from the adverse health effects of air pollution.^2^ However, to be able to best mitigate the impact of air pollution on public health, it is crucial to have reliable, meaningful, open-source, and quantitative air quality data in line with Giles-Corti and group’s assertion “what gets measured, gets done”.^6^ A limited number of governments worldwide carry out routine and relatively widespread air quality monitoring based on networks of reference grade instruments operated using accepted standards and protocols. This approach is expensive to initiate and operate, is logistically challenging, and is limited in terms of expansion for these same reasons.

In relatively high-income countries like the US and the UK, regulatory monitors are typically distributed across urban areas, with one monitoring station covering 100000 to 600000 residents and there are often dozens of regulatory monitoring stations in larger cities.^7^ These regulatory air quality monitoring stations and networks have been instrumental in enabling the scientific and regulatory communities to collect reliable and accurate data, which is essential for developing effective air pollution management and control policies to safeguard public health.^8,9^ The data obtained from these monitoring stations also play a critical role in developing long-term air quality management plans.^8^ These data are used to identify areas where air quality is below identified targets or limits and to determine the individual or general sources of pollution, which can then be targeted with policies to reduce pollution levels and protect public health. By analyzing trends in air pollution data over time, policymakers can determine whether their policies and regulations are effective in reducing air pollution levels and protecting public health.^8^ In contrast, in low- and middle-income countries (LMICs), air quality monitoring is sporadic due to limited logistics, the cost associated with procuring and operating regulatory monitors, and limited local expertise.^10^ Air quality monitoring programs in LMICs where they exist tend to focus on PM_2.5_ monitoring due to three key factors. First, exposure to PM_2.5_ is a key driver for the disease burden associated with outdoor air pollution.^2^ Second, PM_2.5_ is globally recognized as a crucial indicator of urban air quality and is used to establish national air quality standards (see ref (11)). Finally, there are limited resources, capabilities, and expertise in LMICs for running different technologies to monitor other key air pollutants such as gaseous pollutants.^10^

Micro air sensors (defined in this study as smaller air sensors due to their size and minimal logistical demands for installation and operation), or low-cost sensors, have the potential to revolutionize air quality monitoring in urban settings, especially in regions where more traditional monitoring methods based on reference grade instrumentation are sparse or absent. In high-income countries, micro air sensors have been used for various purposes, including raising awareness of air quality issues,^12^ identifying hot spots of atmospheric emissions,^13^ and complementing regulatory monitoring.^14^ These applications have been supported by research institutions, regulatory bodies, and community science organizations, which have validated and calibrated local data from micro air sensors for PM monitoring to support air pollution research and mitigation.^15,16^

One of the main challenges in use of this class of PM sensor as part of regulatory or compliance monitoring is the lack of accepted standard calibration methodologies for micro PM air sensors, which makes it difficult to use the fine scale data for developing site-specific mitigation policies and estimating the health burden of air pollution.^13,14,17−21^ Micro air sensors are also known to respond differently to particles of different size fractions^22,23^ or compositions.^24^ Accounting for these differences requires careful calibration across a variety of particle size and composition regimes, which is challenging in practice.^25^ In addition, there are no specific standards for selecting and using PM sensors to supplement sparsely distributed air quality monitoring stations with relatively poor spatial and temporal resolution air quality data.^19,26−28^ For example, reference stations operated by the Ghana Environmental Protection Agency rely on conventional gravimetric methods for reporting data for PM at six-day resolution, generating ∼5 data points per month (Personal Communication, EPA Ghana). This poor temporal resolution limits our understanding of local trends in PM pollution. Thus, integrating data from micro air sensors is a potentially promising new approach for source identification,^29^ though micro air sensor use has some challenges highlighted above.

In this study, we demonstrate the suitability of high-resolution data from a selected micro air sensor when coupled with a receptor model approach for identifying sources of PM_2.5_ in an urban setting typical of the geographic region to help develop, implement, and track clean air solutions.

## 2 Methods

### 2.1 Study Area

**Figure 1:**
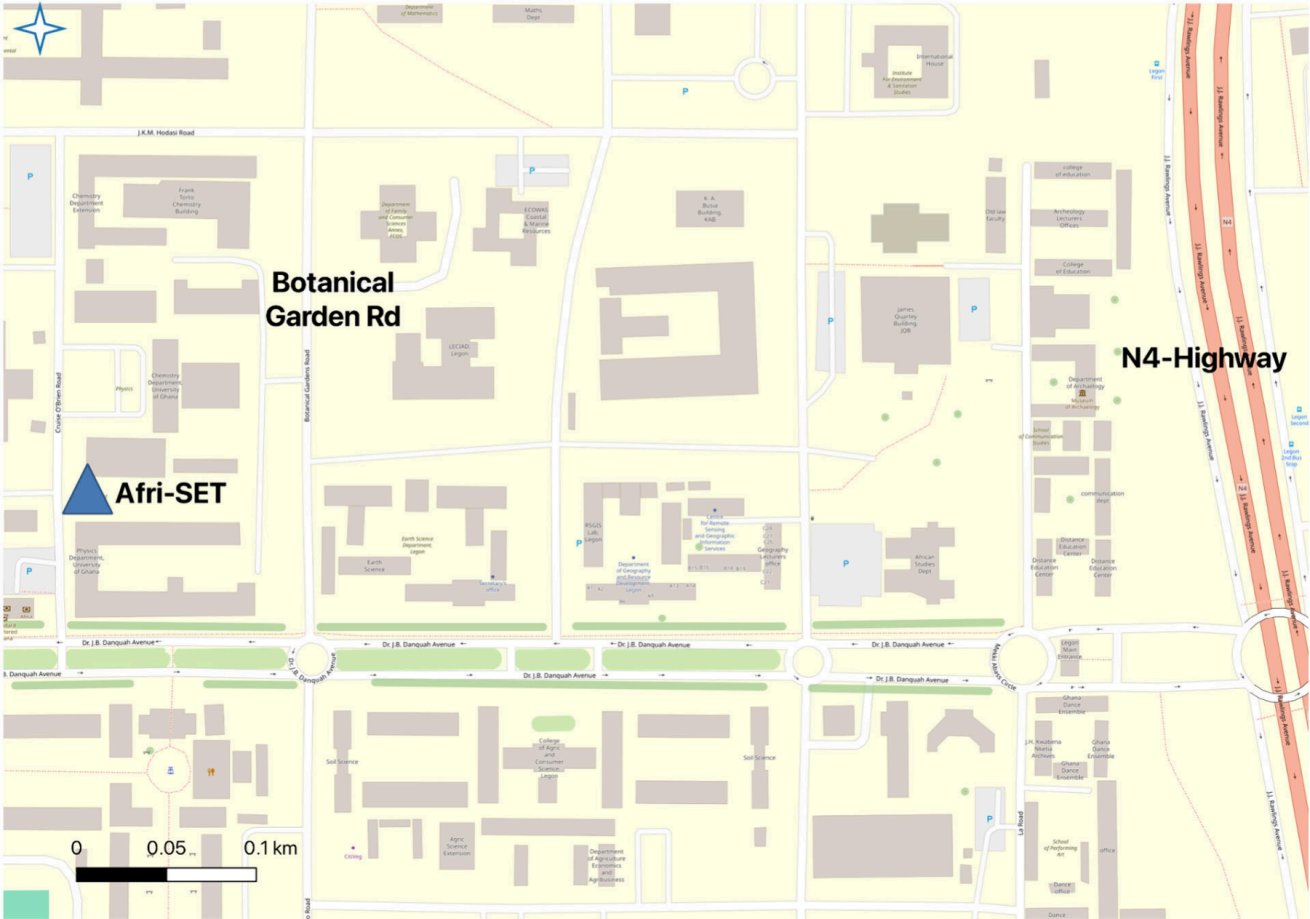
Map showing the study area with the blue triangle labeled Afri-SET indicating the study site highlighting the N4-Highway and Botanical Garden Road developed using QGIS version 3.32.0-Lima.

As shown in previous studies for calibrating micro air sensor data, a crucial factor is collocating the micro air sensors with regulatory or reference grade monitors in the same environment.^14,21,30−32^ Sampling the same air over time generates the paired micro air sensor and reference grade monitor data required for calibration. In this study, the Department of Physics, University of Ghana, Legon (now known as the Afri-SET reference site), was selected as it routinely measures PM with a reference-grade PM mass monitor and is representative of the Greater Accra Region (GAR). An AirGradient Open Air PM monitor was colocated with a Teledyne API PM Mass Monitor at the site for ∼3 months for this case study. The University of Ghana is approximately 8.4 km north of Accra Central and 4.8 km from the N4-Highway South (a major regional route, see Figure 1). The GAR has a population of approximately 5.5 million inhabitants.^33^ Weather patterns across Ghana are not uniform, with significant variation between coastal and maritime influenced zones and more inland areas. The climate of the GAR is characterized by seasonal harmattan winds, which blow from the northern part of the country between late December and February, as well as the southern rainy season that peaks in August and September, with heavy rainfall occurring from April to June. Annual rainfall is 1250 mm in the northern part of the country and 2150 mm in southern Ghana. The GAR climate is tropical, predominantly warm and humid with an annual mean temperature between 26 and 29 °C.^34^ Prevailing climatic conditions in the GAR are typically affected by dry tropical continental winds that mainly originate from the northeast and cross the Sahara. Conditions at the Afri-SET site are characterized by complex sources of PM_2.5_. To the east there is the N4-Highway and to the northeast and southeast of the site is the campus road network, including the Botanical Garden Road that routinely experiences major levels of traffic. On-campus transportation and other background activities such as open burning in the local area are also a factor.

**Figure 2:**
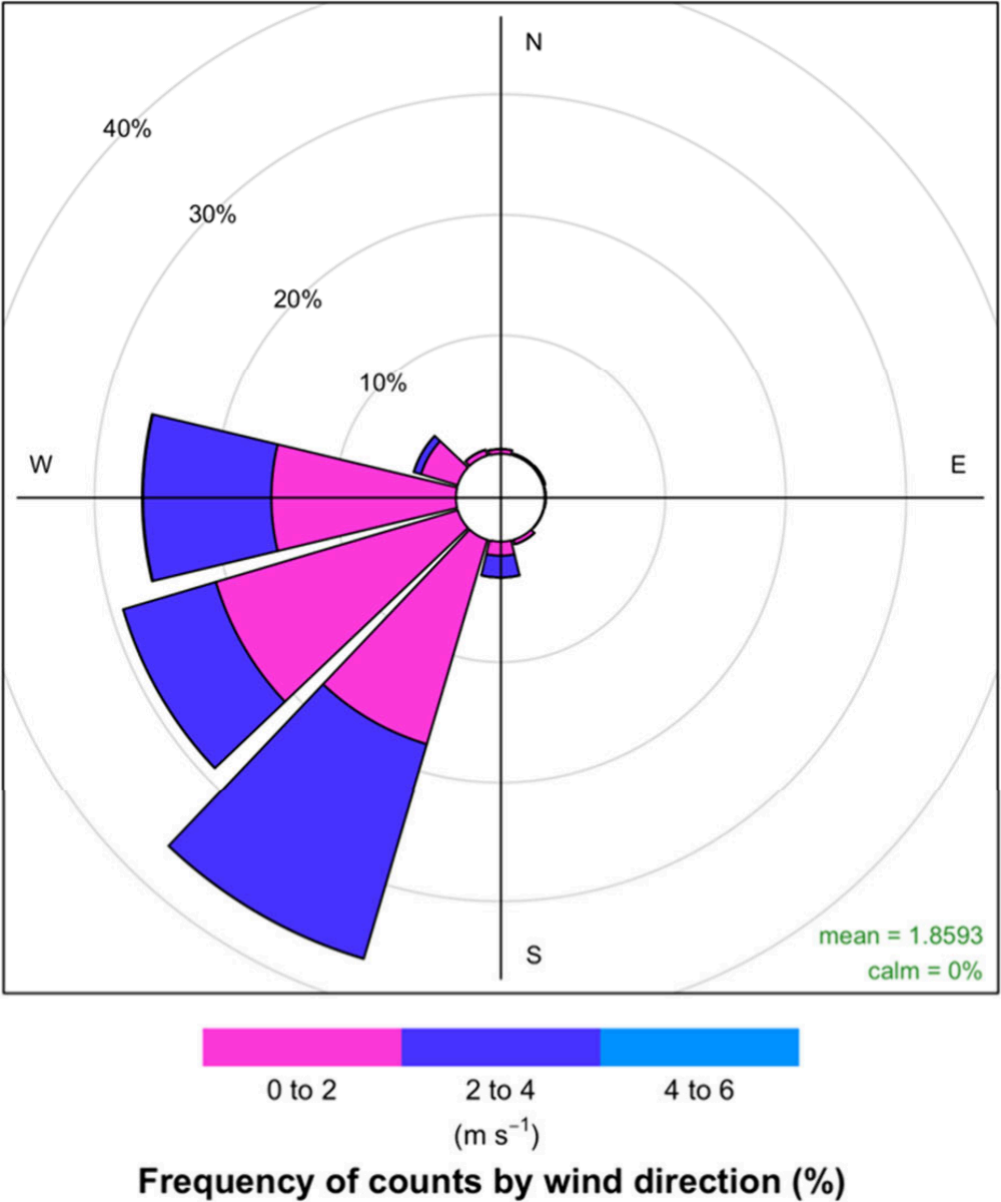
Wind rose plot showing wind speed/direction frequencies for the Physics Department at the University of Ghana (Afri-SET), where the study was conducted for the period of deployment, based on hourly wind data from the weather station at the site.

A wind rose plot presented in Figure 2 shows that the dominant wind direction at this site for the period of the deployment was almost exclusively from the southwest sector.

### 2.2 Instrumentation and Data Acquisition

**Figure 3:**
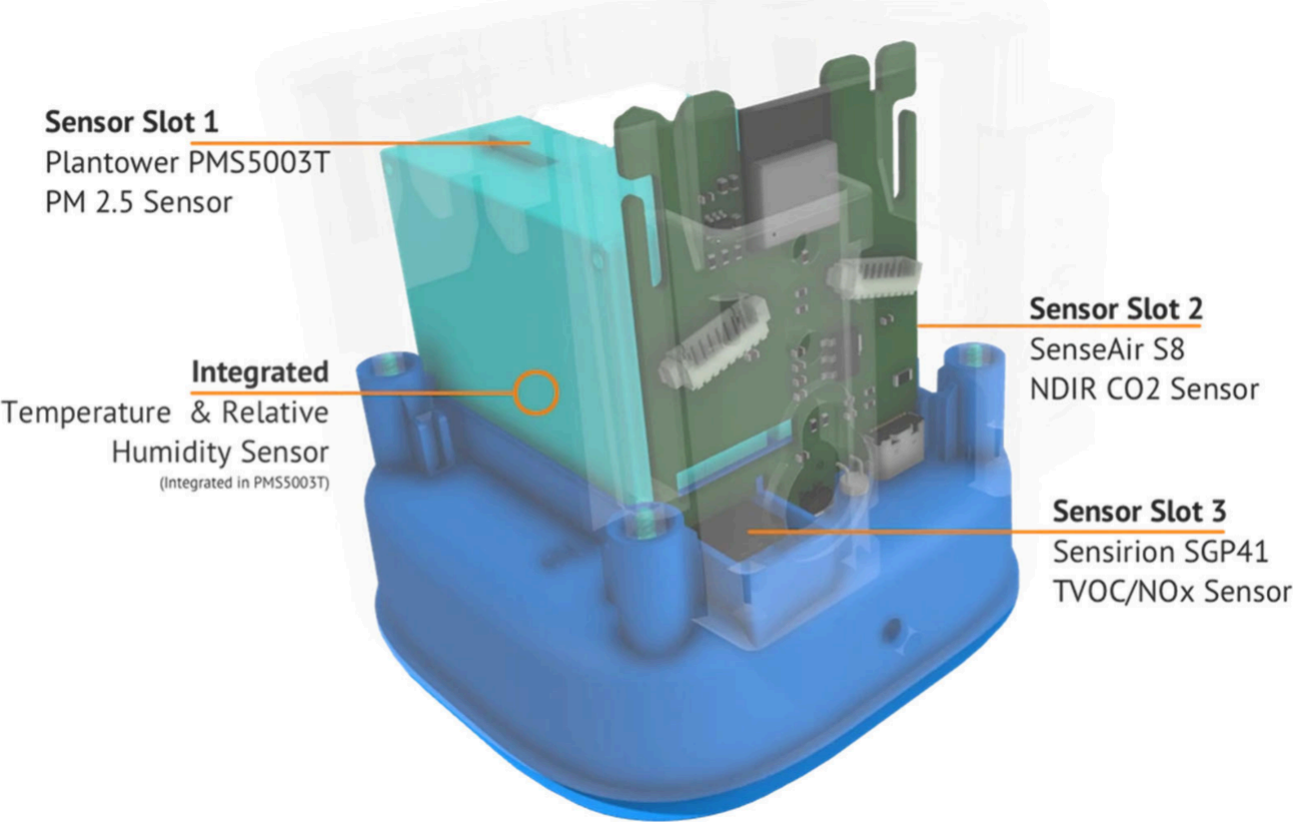
AirGradient Open Air PM_2.5_ Monitor. Image adapted from AirGradient.

The micro air sensor used in this study is the AirGradient Open Air PM (AirGradient, Chiang Mai, Thailand). This micro air sensor was deployed at the Afri-SET reference site following a protocol similar to that used by the Raheja group.^32^ It was collocated with an Afri-SET reference PM Mass Monitor (Teledyne API, San Diego, California, USA). Both the micro air sensor and Teledyne API PM Mass Monitor were placed at a height of approximately 5 m above ground. The AirGradient Open Air PM uses a Plantower PMS5003T sensor element and reports data via the Open Air platform (AirGradient, Manual). Figure 3 shows how the PMS5003T is mounted within the AirGradient Open Air PM monitor. The PMS5003T is widely used by micro air sensor manufacturers (e.g., QuantAQ, AirQo, Clarity Movement, and PurpleAir). The PMS5003T uses light scattering to measure PM (i.e., by employing a laser to estimate particle concentrations with manufacturer-defined algorithms, which convert the scattered light into particle concentrations). A number of manufacturers using the PMS5003T report data for PM_1_, PM_2.5_, and PM_10_; however, emerging evidence shows that the PMS5003T is only suitable for measuring PM_2.5_.^22,35^ Current best practice for use of micro sensors under real world conditions is to apply a domain specific calibration to improve the data representativeness (e.g. refs (35−38)). To generate calibration factors, sensors are collocated with an accepted reference instrument that is being operated according to appropriate operational protocols (e.g., Federal Reference Method and/or Federal Equivalence Methods) for a representative period (where representative refers to both the expected range of pollutants as well as climatological conditions). Uncorrected PM_2.5_ data from the micro air sensor are then compared with the reference data and an analysis is undertaken to generate comparative statistics and correction factors which, when applied to the uncorrected data, bring them into line with the reference data. Methods to generate these factors include regression analysis and increasingly machine learning tools (see e.g., refs (13, 14, 25, 30, and 37)). As a part of this process, a correction to account for temperature and humidity can be applied based on locally reported temperature and relative humidity.^13,14,25,30,37^ The reference PM instrument has the ability to provide 1 min time resolution data but, as operated at the Afri-SET site, reports hourly processed data via the Ghana EPA.

### 2.3 Data Cleaning and Calibration

Standard quality control and assurance measures were employed to clean the 5 min resolution data from the AirGradient Open Air monitor. Following the same quality control procedure as shown in ref (30) for Plantower devices, all zeros and spurious values above 1000 μg m^–3^ were removed and we ensured that the time stamp was representative of the local time zone. A common time stamp was selected for calibration with the two sets of data in the same resolution. Simply put, the 5 min data were converted into hourly average data to match the resolution of the Teledyne API PM Mass Monitor PM_2.5_ data sets. For this study, this resulted in 2544 paired data points over the 107-day deployment.

Prior studies on micro air sensors in Ghana have used different types of micro air sensors and multiple types of correction factor algorithms with varying degrees of complexity depending on the objectives of the study (e.g., the Gaussian Mixture Regression model by refs (30 and 39)) and inbuilt calibration protocols (e.g., refs (22 and 40)). Previous studies, such as that in ref (41), have demonstrated that multiple linear regression (MLR) is a useful tool for improving raw PM_2.5_ data quality and can be applied to improve micro air sensor data to meet regulatory recommendations (e.g., refs (14, 21, and 30)). Its application for improving air sensor data is suitable for extracting source features of pollutants.^42^ This is particularly important in technologically lagging environments with limited regulatory monitoring mechanisms and expertise. Thus, a MLR model was used to calibrate the AirGradient Open Air PM_2.5_ data sets with internal temperature and relative humidity measurements from the same instrument. The linear equation developed to calibrate the reported data is
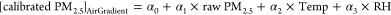
1where α_0_ = intercept from the statistical summary of the model, α_1_ = coefficient of raw AirGradient PM_2.5_ data, α_2_ = coefficient of temperature, and α_3_ = coefficient of relative humidity.

We withheld a randomly selected 20% of the reported data to test and validate the correction model while using 80% to develop the model by identifying suitable values for the coefficients using a linear regression package implemented in the R-programming language and environment such that the linear model summary generated values for each of the coefficients α_0_, α_1_, α_2_, and α_3_. We then present a case for the 3 sets of data (raw, calibrated, and reference grade) to identify the potential sources of PM_2.5_ as shown in the rest of the analysis.

### 2.4 Data Analytical Approach

We based all analysis on the “openair” package for air pollution data analysis using the functions windRose, timePlot, scatterPlot, timeVariation, polarPlot, calendarPlot, and timeProp for wind rose, time series, scatter, daily variation, bivariate polar, calendar, and temporal variation plots, respectively,^43^ in the R-programming language and environment version 2024.09.0+375. For source apportionment, we employed a receptor model.^43^ Source apportionment studies using this approach are not new,^44,45^ but this approach with air sensor data is relatively new. Reference (29) attempted to identify sources of PM in an urban setting in Ghana using high temporal resolution data from air sensors. A recent study in Birmingham, UK has also shown that micro air sensor data is useful for inferring sources of PM in a quarry setting.^46^ We introduced a relatively new concept using the PM_2.5_/PM_10_ ratio in bivariate polar, scatter, and count plots as shown in refs (47 and 48) to provide insights into the constituents of the reported PM_2.5_ using calibrated PM_2.5_ data and PM_10_ measurements from the Teledyne API PM Mass Monitor.

## 3 Results

### 3.1 Linearity and Precision of the Air Sensor

By comparing the AirGradient and T640 PM_2.5_ data sets, we observed that the AirGradient overestimated the PM_2.5_ concentrations by 34% with a mean absolute error (MAE) of 5.44 μg m^–3^ and *r*^2^ of 0.85. The following equation was developed using the MLR model to improve out of the box PM_2.5_ values from the AirGradient monitor, as discussed in Section 2.3.
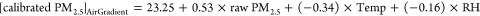
2

**Figure 4:**
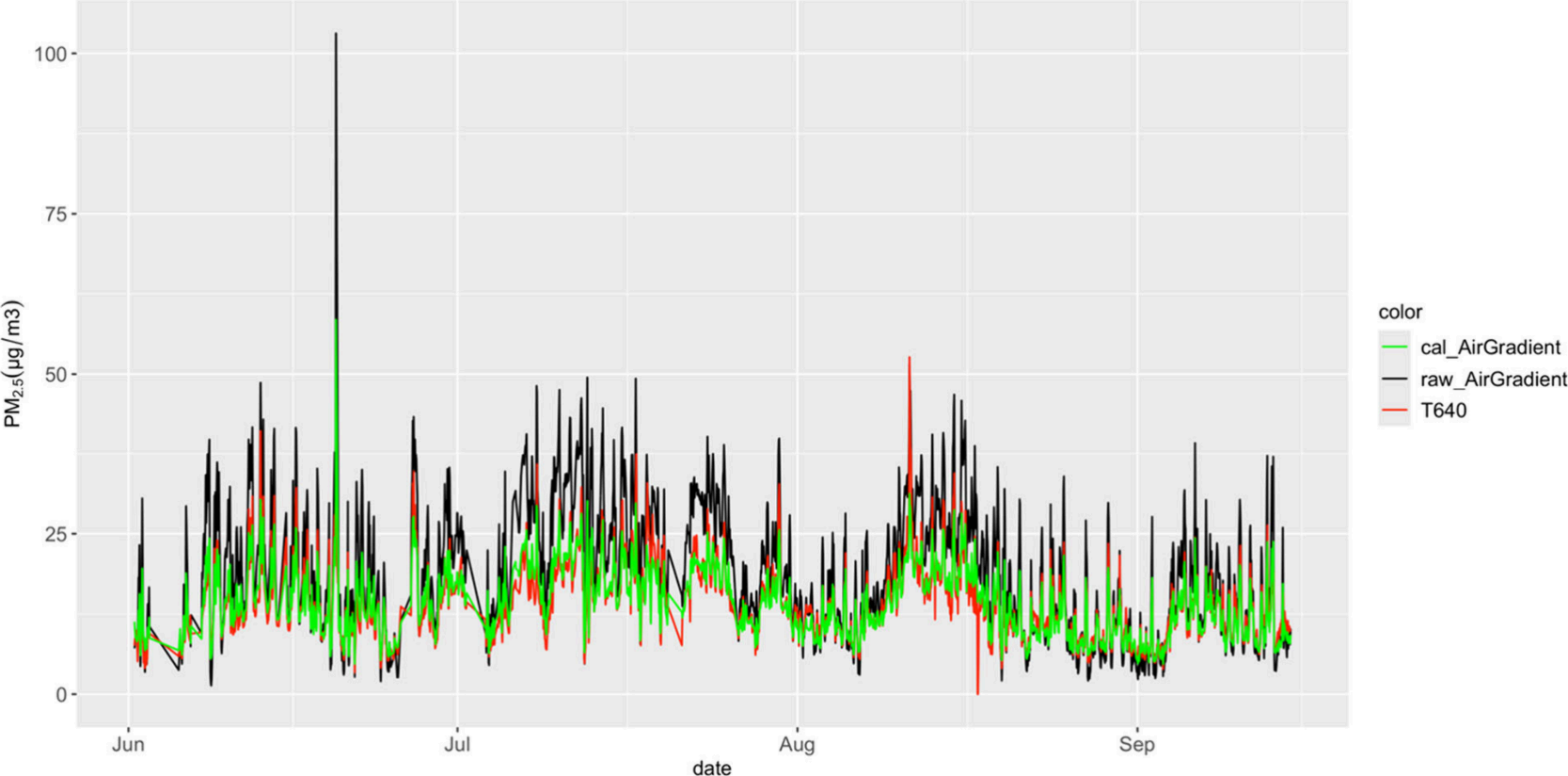
Time series plot for PM_2.5_ showing AirGradient raw (black), calibrated (green), and T640 (red) on hourly data sets.

Using this MLR model, we calibrated the AirGradient PM_2.5_ data and achieved an improved MAE to 1.43 μg m^–3^ (Table 1). The nearness of the MAE to zero indicates the accuracy of the model used for calibrating the PM air sensor data. This observation compounds the growing body of evidence on the need for calibration when using air sensor data in these types of environments, particularly since environmental agencies are now turning to these types of approaches for estimating air pollution. Reported mean values were 19.03 μg m^–3^ for raw air sensor data and 14.17 μg m^–3^ for calibrated air sensor and reference-grade data, representing a 34% overestimate by the raw data. Figure 4 shows that calibrated and reference data agree well with each other, and raw overestimates but also follows the same trends, as reflected in the high *r*^2^.

**Table 1:** Regression Model, Average Time, *r*^2^, and MAE Statistics for Raw and Calibrated PM_2.5_ Data

data type	model	average time (h)	*r*^2^	MAE (μg m^–3^)
AirGradient_raw	none	1	0.83	5.44
AirGradient_cal	MLR	1	0.85	1.43

**Figure 5:**
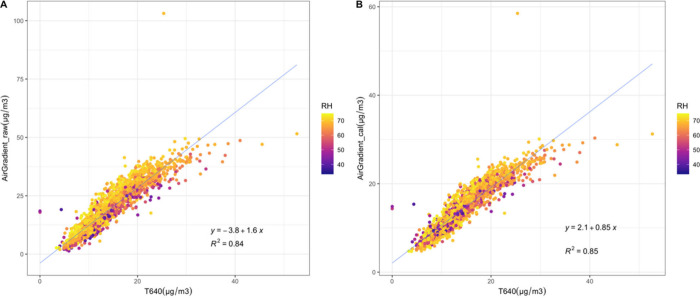
Scatter plot for PM_2.5_ as a function of RH for raw (A) and calibrated (B) data against T640 data.

Also, we observed that the impact of relative humidity on the observed PM_2.5_ was significant at a higher relative humidity. A scatter plot is presented in Figure 5 for the raw (Figure 5A) and calibrated (Figure 5B) data against the reference grade data sets. Both the raw and calibrated data have a strong linear relationship with the reference data. The AirGradient (raw) overestimated PM_2.5_ at higher relative humidity (>70%), which was largely accounted for using the calibration factor in Figure 5B.

### 3.2 Sources of PM_2.5_

**Figure 6:**
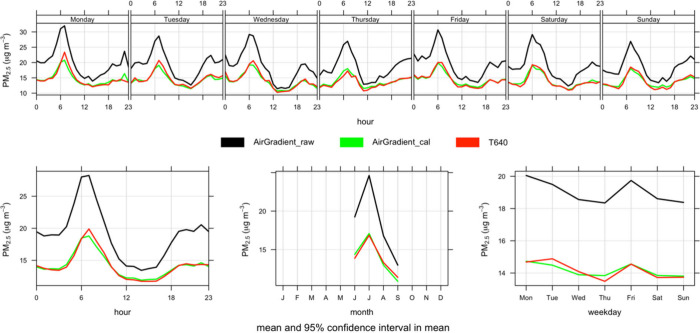
Daily variation of PM_2.5_ using raw (black), calibrated (green), and T640 (red) data sets.

Fine scale data from air sensors are useful for understanding trends in local pollution that have previously been unachievable. We employed a timeVariation function as a source feature tool to understand the impacts of anthropogenic activities on the observed PM_2.5_ data. We observed a similar pattern in peak periods with caveats when comparing the raw to the calibrated and reference-grade PM_2.5_ data sets. It was observed that PM_2.5_ pollution was influenced by human activities such as vehicular emissions and biomass burning, considering background activities and hours of the day of observed peaks. For example, we observed hourly peaks of 20 μg m^–3^ around 07:00 h for calibrated and reference grade data but ∼32 μg m^–3^ for the raw data (Figure 6). This was due to the overestimation as flagged above, but the raw data do provide a clue on sources, especially by linking the observed concentrations to hours of the day and prevailing human activities.

Daily variation for PM_2.5_ using the high-resolution data showed peaks in concentration associated with the morning rush hour around 07:00 h and evening rush hour at around 18:00 h (left bottom panel, Figure 6). We also observed that the concentrations do not drop overnight, which could be due to prevailing meteorological conditions and potentially cooking and/or biomass burning. Reduced background activities between 12:00 h and 15:00 h caused drops in the reported levels (left bottom panel, Figure 6). Monthly, lower concentrations were observed in September due to the heavy rains which might have washed down PM_2.5_, though this was only for the first 15 days of the month (middle bottom panel, Figure 6).

**Figure 7:**
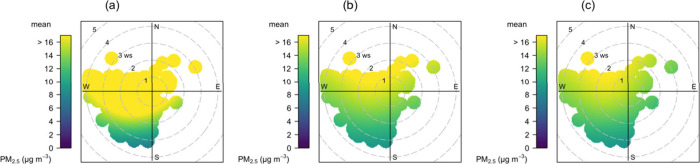
Hourly bivariate polar plot for PM_2.5_ using AirGradient raw (a) and calibrated (b) and T640 (c) data sets.

Also, we adapted bivariate polar plots to identify sources of PM_2.5_, which provides a clear graphical representation of the observed PM in relation to wind speed and direction. Graphically, we observed that the NW quadrant (Figure 7) is a major source of PM_2.5_, which comprises campus road networks and commercial activities where food cooking and vending is based on a mixture of energy sources (charcoal, liquified petroleum gas) and open burning. Also, we observed that the highest concentrations in the raw data are linked to the SW wind direction but do not appear in the calibrated data. Although an overestimation of concentrations in the raw data was observed, this indicates that the overestimate is not purely systematic but has some dependence on wind direction, possibly due to different meteorological factors (e.g., high humidity) being associated with certain wind directions. This is an important confounding factor to note when using uncalibrated micro air sensor data to conduct source identification. A bivariate polar plot of the reported data is presented in the Supporting Information (Figure S1, at the levels of relative humidity, and Figure S2, at the levels of temperature). Relative humidities were grouped into four levels; first 33.4–56.7% (PM_2.5_ was mostly from NW, W, and S for the raw and NW for the calibrated and reference-grade), 56.7–66.8% (PM_2.5_ was mostly from all quadrants for the raw and NE, NW, and SW for the calibrated and reference-grade); 66.8–69.6% (PM_2.5_ was mostly from all wind sectors for the raw except that toward the W sectors and higher sources were observed at winds speeds >3 ms^–1^; a similar observation was made for the calibrated and reference-grade data but the graphical representation shows a smaller margin toward the SW), and 69.6–74.9% (higher local sources for PM_2.5_ from all wind sectors with a slightly bigger margin toward the N as compared to the observations at 66.8–69.6% for the raw data set; NE, N, and NW sources for the calibrated and the reference-grade data showing lower levels, below 12 μg m^–3^ from the NW quadrant) (Figure S1). In Figure S2, similar observations were made except in the opposite such that the sources at lower temperature levels, e.g., 21.5–24.3 °C, corresponded to the observations at higher relative humidity levels, i.e., 69.6–74.9%, which is expected due to the inverse relationship between temperature and relative humidity in atmospheric observations.

**Figure 8:**
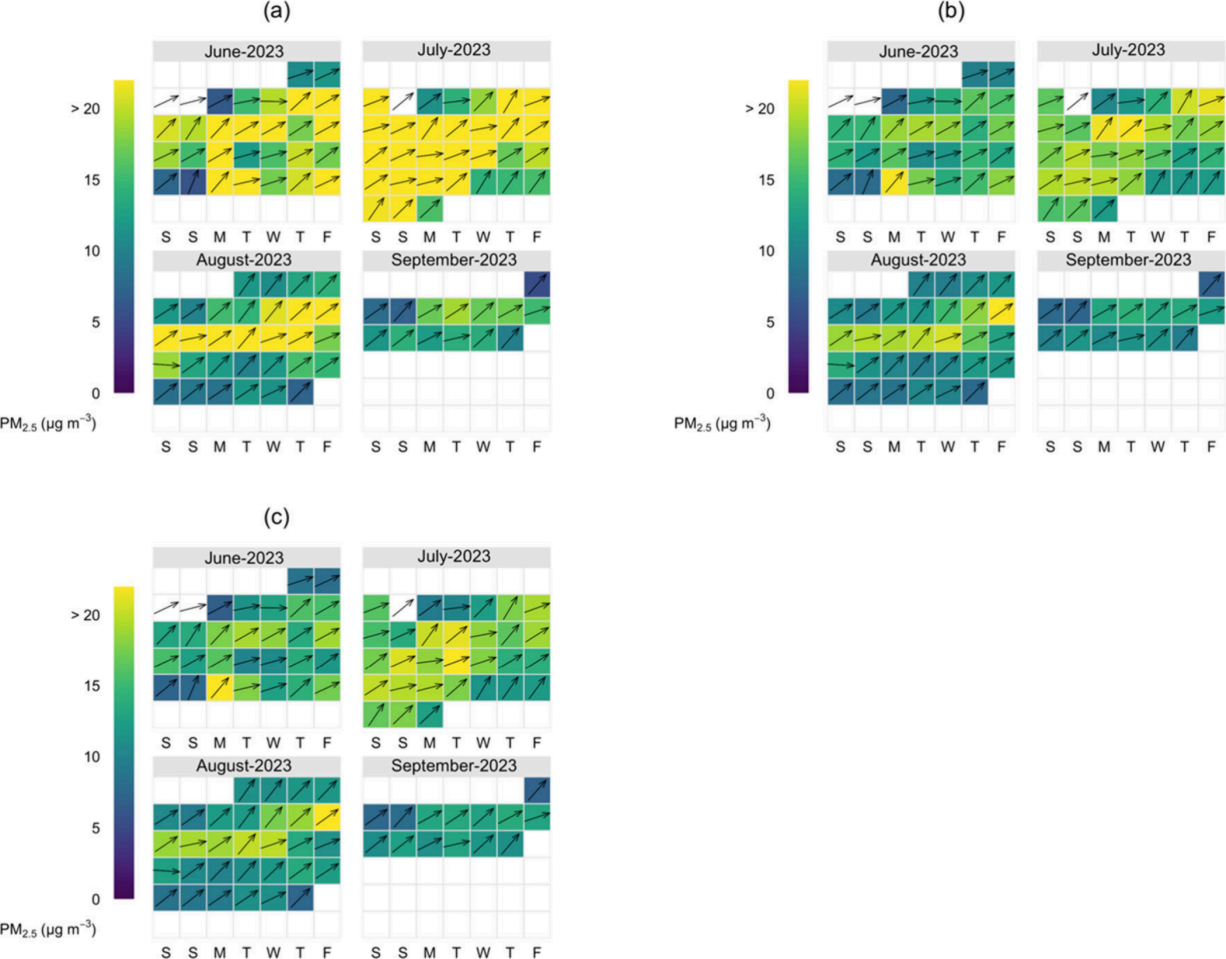
Calendar plot for PM_2.5_ as a function of wind direction showing AirGradient raw (a) and calibrated (b) and T640 (c) data sets.

Further to the above analysis, we employed the calendar plot to provide a graphical picture of wind direction on the observed daily PM_2.5_ concentrations. These plots revealed common wind direction for the 3 sets of data with varying mean values. Daily average winds were exclusively from the W or SW; therefore, it was impossible to identify other wind directions which might have contributed to the PM_2.5_ pollution for this period at the site using this daily average data alone (Figure 8).

**Figure 9:**
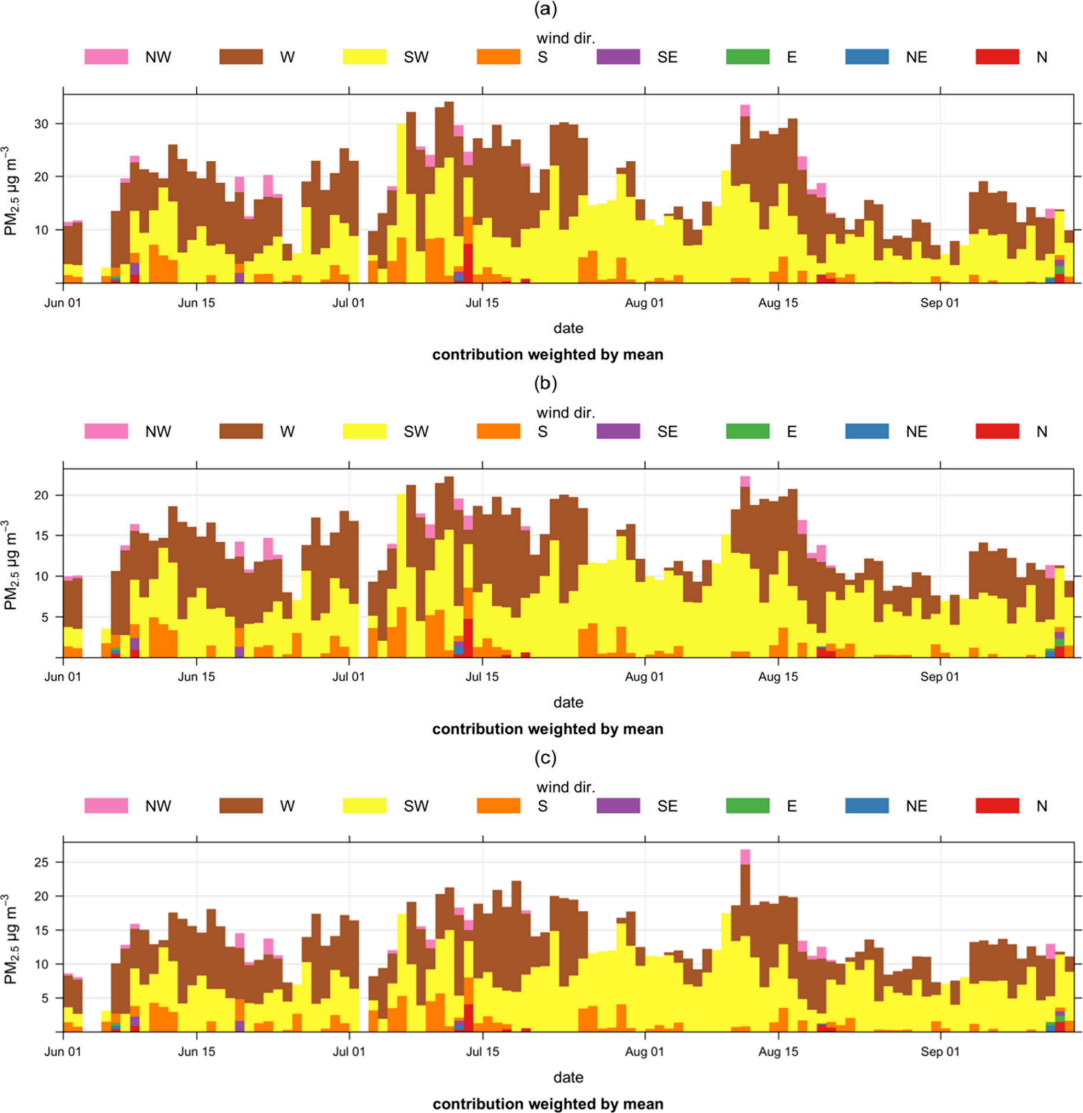
Time series plot for PM_2.5_ as a function of wind direction raw AirGradient (a), calibrated AirGradient (b), and T640 (c) on hourly data sets.

To understand the impacts of wind direction on hourly observations, we plotted the observed PM_2.5_ levels (3 sets of data) as a function of wind direction. We observed that W, NW, and SW winds contributed to higher PM_2.5_ but NW winds were patchy and associated with higher concentrations (35 μg m^–3^ for the raw and 23 μg m^–3^ for both the calibrated and reference-grade PM_2.5_ data sets) (Figure 9). This was followed by W winds (35 μg m^–3^ for the raw and 23 μg m^–3^ for both the calibrated and reference-grade PM_2.5_ data sets) and SW (30 μg m^–3^ for the raw, 20 μg m^–3^ for the corrected, and 18 μg m^–3^ for the reference-grade PM_2.5_ data sets) (Figure 9). It is noteworthy that these observations were not shown in the bivariate polar and calendar plots, which shows the limitations of relying solely on bivariate polar and calendar plots for extracting the source features of pollutants. Though the dominant wind source was from the SW direction (Figure 2), higher observed concentrations were associated with the NW winds, indicating a potential major source in this direction (local), which were influenced by a mixture of background activities including vehicular emissions, windblown dust, and biomass burning. This plot also revealed that there were multiple sources of PM_2.5_ pollution at this site, but identified sectors with higher sources need to be further investigated.

**Figure 10:**
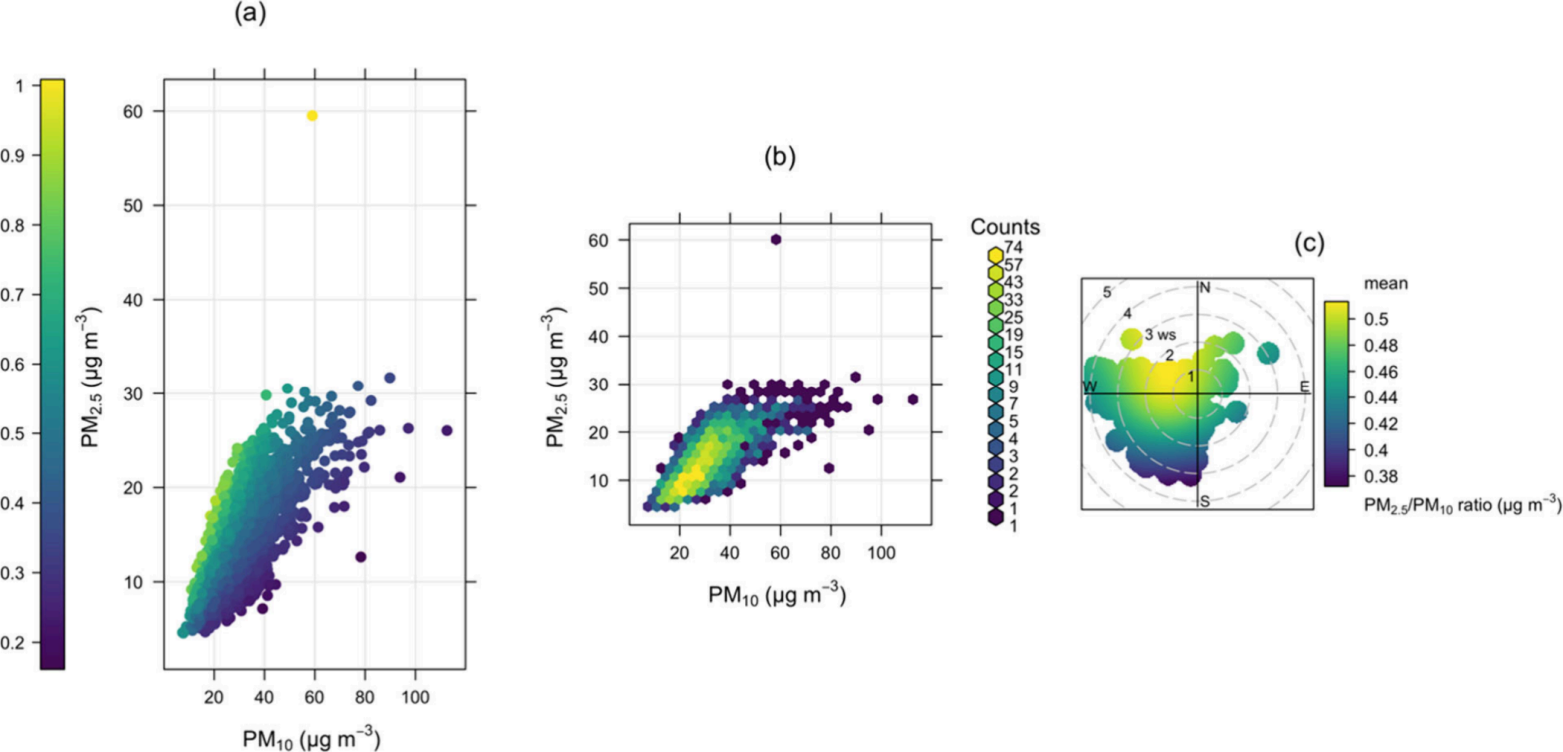
Scatter plot of PM_2.5_/PM_10_ ratio (a), particle count (PM_2.5_ vs PM_10_) (b), and bivariate polar plot for PM_2.5_/PM_10_ (c) ratio in color code on hourly data for June 1 to September 15, 2023.

Using PM_10_ data from the Teledyne API PM Mass Monitor, a scatter plot using the hourly data of PM_2.5_ against PM_10_ with the PM_2.5_/PM_10_ ratio is presented in Figure 10a showing three main categories of ratios: ratios in blue (i.e., <0.3), green (i.e., between 0.3 and 0.9) and yellow (i.e., between 0.9 and 1). In Figure 10b, the scatter plot of PM_2.5_ and PM_10_ concentrations is drawn based on the ratios. The PM_2.5_/PM_10_ ratio in the bivariate polar plot in Figure 10c reaffirms a potential source of fine aerosol in the NW wind sector from vehicular activities and biomass burning.

## 4 Discussion

We used a 15-week data set to compound emerging evidence on the usefulness of micro air sensor data for source identification of PM_2.5_ in an environment where this information will be more readily available. The analysis presented in this work is unachievable using conventional gravimetric monitoring in Ghana, which generates only 5 data points per month depending on the availability of consumables. It is however important to note that, as demonstrated in previous studies and highlighted in the introductory part of this study, the use of micro air sensors does come with some disbenefits, specifically the impacts of temperature and relative humidity on the reported data, hence the need for domain-specific calibration (see previous studies from this site and similar environments^30,31^). Reference (29) published the first-ever work on low-cost source identification using micro air sensor data in Africa at an urban area in Central Ghana with relative concentrations of PM_2.5_. A recent publication from Westervelt and his group^42^ re-echoed this evidence that micro air sensor data are useful for tracking sources of air pollutants and^46^ also inferred sources of PM_2.5_ at a quarry using fine scale air sensor data. In this specific work, we initially evaluated the performance of an AirGradient micro air sensor in a tropical environment with complex and high sources of PM_2.5_. We then make a case to support emerging evidence on micro air sensor use for source identification of pollutants, PM_2.5_ in this case. The results have shown that wind speed–direction data can provide a useful tool for inferring sources of PM_2.5_ when combined with high-resolution micro air sensor data. A further investigation of the identified wind sectors would generate added value for PM_2.5_ emission control at and around the urban environment at the University of Ghana. This work contributes to the growing body of knowledge on the use of high resolution data from air sensors for cost-efficient identification of sources of air pollution in major parts of Africa with limited air quality monitoring capabilities. It is a useful reference for developing, implementing, and tracking air pollution mitigation strategies in these types of environments by regulatory bodies and other relevant stakeholders.

### 4.1 Linearity and Precision of the Air Sensor

The AirGradient monitor overestimated PM_2.5_ measurements by 34% but the trends follow the same pattern as compared to the referenced grade reported data. Similar findings were observed in previous studies using air sensors in Africa.^30,31,42^ This shows that raw air sensor data are useful for understanding local PM trends, but to support local mitigation strategies, community engagement, and emission source identification, data improvement via local collocation with reference instruments is required.

### 4.2 Sources of PM_2.5_

Reference (49) showed that PM pollution in Accra neighborhoods is linked to charcoal and fuelwood burning and traffic. In this study, bivariate polar, calendar, time series, PM_2.5_/PM_10_ ratio scatter, and count plots as a function of wind speed–direction were used to identify the sources of PM_2.5_ at the University of Ghana. The aim was to verify if relative measurements from air sensors could provide useful insights for source feature extraction, as previously shown in ref (29), which is linked to background activities as echoed by ref (49). We found that emissions were mainly from the NW sector (vehicular emissions from campus road networks and commercial activities, which includes use of solid fuel and burning of waste). These findings align with past studies using similar sensors,^40^ although long-term data would be needed for further verification at the University of Ghana.

It is worth noting that protocols for assessing air sensor data for source identification depend on the investigator’s purpose, but previous studies have shown that a ±50% data quality from air sensors is sufficient for extracting source features of atmospheric pollutants in highly polluted environments.^50,51^ The findings as presented in this study using the raw, calibrated, and regulatory data to identify the sources of PM_2.5_ pollution using the receptor model compounds emerging evidence on the usefulness of micro air sensor data to support source feature extraction, a low capital cost approach for source identification (e.g., refs (29, 42, and 46)). The calendar plot was used to achieve this objective and indicated that high PM_2.5_ pollution was driven by southwesterly winds. A limitation of the calendar plots presented is that, for the daily average data, there is little variability in wind direction (as noted previously, throughout the study period, winds tended to originate from the southwest). This makes it difficult to distinguish the potential influences of different sources. On the other hand, the high temporal frequency data available from the air sensors allows for finer distinctions to be made based on more frequent changes to wind speed and direction, as evidenced by the polar plots and time series plots in wind direction. However, micro air sensors do not measure wind speed–direction, indicating the need for site specific wind component data using auxiliary instrumentation or modeled data as demonstrated in ref (29). This study also highlighted that the use of polar plots for understanding the sources of pollutants has limitations and that understanding meteorological factors, such as wind direction and speed, is crucial for air pollution management and control. To account for this, we used the PM_2.5_/PM_10_ ratio as shown in ref (47) to provide core insights into the potential components of the observed PM. The results demonstrate the potential usefulness of micro air sensor data for managing and controlling air pollution in LMIC with limited air quality monitoring capabilities at low cost. Furthermore, it was observed in this study that the identified source of PM pollution was slightly different across all data sets in the bivariate polar plot noting the difference in the raw hourly data as compared to the calibrated and reference-grade data sets but similar in the time series plot in wind direction. This shows that relative concentrations of PM_2.5_ measured from micro air sensors can be used to develop and track mitigation strategies for air pollution management and control. While the AirGradient monitor is not filter-based to support speciation, the approaches applied here give stakeholders, especially the environment agencies in Africa that are now turning to lower-cost air quality monitoring approaches, a toolkit for further investigation of background activities.

### 4.3 Hourly Bivariate Polar Plot Sources and Time Series in Wind Direction of PM_2.5_

A limitation of the polar plot is that the graphical identification of source of pollutants is influenced by the capabilities of the investigator and these plots only group mean concentrations by wind speed and direction,^43^ making it difficult to specifically match wind sectors with sources of pollutants. To provide a clearer picture that is only achievable with high-resolution data, we introduced the time series plot as a function of wind direction (Figure 9). For example, in ref (29), a cluster analysis was introduced to group sources from the same wind speed–directions to augment the findings in the bivariate polar plots. By way of observation, multiple and the same wind directions contributed to the reported PM regardless of the data quality but concentrations varied. Comparatively, in the bivariate polar plot for example, higher sources were associated with the NW, W, and SW winds for all sets of the data (Figure 7). However, in the raw data sets, the SW wind speed–direction was noticeable as compared to calibrated and reference-grade data sets (Figure 7). The time series plot in wind–direction shows sources are similar but higher concentrations were associated with NW sector followed by the W and SW. The contribution from the NW were however patchy though associated with high concentration indicating a major source; winds from the W were dominant and similar to those from SW but with concentration below 30 μg m^–3^ for the raw, 20 μg m^–3^ for the corrected, and 18 μg m^–3^ for the reference data sets. This finding echoed previous findings on the usefulness of fine scale relative concentrations from micro air sensors to identify sources of pollutants in urban settings. This is however not achievable with the filter-based monitoring regime currently operated by the Ghana EPA, since there are only ∼5 data points per month. However, filters do provide a useful tool for source apportionment following laboratory analysis, which can support the findings in this study if combined. The laboratory analysis of these filters requires expertise and is associated with high operational costs, making them expensive to run. In essence, micro air sensors provide a low-cost alternative, where the higher time resolution allows for the detection of shorter-lived sources or of sources from wind directions which are only prevalent for a short part of the day.

Further to the above, in many cases, it will not be possible to perform a local calibration of the micro air sensors, and so examining and emphasizing the advantages and limitations of the raw versus calibrated sensor data can be valuable for those trying to replicate this work. In Figure 6, the hourly, daily, and monthly patterns are basically the same in the raw data as in the calibrated data, just with a different magnitude; this might indicate that even uncalibrated data could be useful for distinguishing between local sources with likely short-term impacts and more regional sources that have longer-term impacts or for comparing the relative impacts of sources at different times of the day. However, in Figure 7, the uncalibrated data show the highest concentrations associated with winds from the SW, while the calibrated data do not; this is potentially a major limitation of using uncalibrated data when relying only on bivariate polar plots using raw values of air sensors for source identification.

### 4.4 PM_2.5_/PM_10_ Ratio

The ratios below 0.3, represented in blue in Figure 10a,c, indicate that PM_10_ is the primary pollutant originating from windblown and resuspended dust due to traffic. A higher ratio of PM_2.5_/PM_10_ (>0.5), represented in yellow in Figure 10a,c, shows a higher fraction of fine (PM_2.5_) than of coarse (PM_2.5–10_) mass which is either emitted from direct burning of biomass or vehicular emissions or by the reactions of nitrogen oxides and sulfur oxides with oxidants such as OH radicals and ozone to form secondary nitrate and sulfate aerosols, considering the background activities.^52^ Most of the data represent a ratio of about 0.5, with a typically strong correlation between PM_2.5_ and PM_10_ (Figure 10b). Since micro air sensors are not filter-based but can generate fine scale PM data as highlighted in the sections above, the PM_2.5_/PM_10_ ratio in scatter, particle count, and bivariate polar plots using the concentrations of the reported PM_2.5_ (calibrated) and PM_10_ (e.g., from the Teledyne API PM Mass Monitor) is a useful model to classify sources of the reported PM tied to the wind speed–direction and background activities. This is useful for signals linked to the potential components of the reported PM classification. Existing works have shown that PM_2.5_/PM_10_ ratios >0.5 signify sources of particulate matter characteristic of fine aerosols and secondary particulates: namely, NO_3_^–^, NH_4_^+^, and organics. In other words, lower ratios are indicative of coarse particles.^53,54^ The suitability of distinguishing between PM_2.5_ and PM_10_ sources using this approach is echoed in ref (47). This is useful and applicable for extracting source features of PM_2.5_ with fine scale data from micro air sensors if combined with PM_10_ measurements. By way of demonstration, we used the calibrated PM_2.5_ and the Teledyne API PM Mass Monitor PM_10_ data from the same site because the AirGradient Open Air micro air sensor is not suitable for measuring PM_10_ as mentioned in the introductory part of this work. It is noteworthy that the PM_2.5_/PM_10_ ratio analysis presented in this work is unachievable without the PM_10_ data from the reference grade Teledyne PM Mass Monitor data. However, other micro air sensor technologies suitable for reporting PM_10_ data such as those offered by QuantAQ which combine different technologies (i.e., Plantower and Alphasense OPC-N3 for PM_2.5_ and PM_10_, respectively) may provide this valuable information for these types of analysis. Per the background activities at this study site for this period, we infer that vehicular emissions and biomass burning are major sources of observed PM_2.5_. This information is desirable in major parts of Africa for clean air solutions considering the increasing use of different micro air sensor technologies for air quality campaigns. Also, the capability of using fine scale micro air sensor data for understanding local sources of PM_2.5_ are useful for regulatory bodies to mitigate air pollution in these types of environments.^48,55,56^ As mentioned, lower ratios point to mainly natural sources such as sand, resuspended dust, and long-range transport (windblown) of dust, a major source of air pollution in Africa and linked to harmattan in Western Africa.^30^ As noted in ref (57), NO_3_^–^ is associated with sources such as soil dust, coal combustion, shipping emissions, sea salt, industrial emissions, biomass burning, and vehicle emissions.

## 5 Conclusions

We compound emerging evidence on the usefulness of air sensor data to support air pollution mitigation in environments where this information will be more desirable. We first compared raw PM_2.5_ data from a micro AirGradient PM monitor to a reference grade monitor Teledyne API PM Mass Monitor T640. The AirGradient overestimated PM_2.5_ values with an *r*^2^ of 0.83 and mean absolute error (MAE) of 5.44 μg m^–3^. We improved the raw data using an MLR model, reducing the MAE to 1.43 μg m^–3^. We leverage the 3 sets of reported PM_2.5_ data (raw, calibrated, and reference grade) to provide insights into PM_2.5_ sources. The results show that out-of-the-box measurements are useful for source identification, with caveats highlighting the need for calibration. Due to the complexity of the site and the limitation of air sensors, we were only able to link the observed dominant wind speed–directions to prevailing background activities such as vehicles and biomass burning from dominant northwesterly winds. This study has shown that the raw high-resolution data from air sensors are useful for source identification and relying on bivariate polar plots has some degree of limitation, especially if these are carried out in an environment with no capabilities of calibrating the air sensor data. To account for this, we recommend a time series plot as a function of wind–direction as shown in this study. We also observed multiple sources at this site for this period, although concentrations vary from each wind sector. We have shown in this study the usefulness of a simplified calibration methodology for improving out-of-the-box measurements from low-cost PM air sensors to provide meaningful insights for source identification. We recommend long-term data collection at this site to provide an overview of long-term wind component data on the distribution of PM_2.5_. Furthermore, in this study, we only make use of hourly data from the micro air sensors, as this is the time resolution for which reference data were available for calibration. However, the sensors are capable of higher (5 min) temporal resolution, which might provide further insights into local sources with short-duration impacts. Further study is needed to evaluate the usefulness of such even higher temporal resolution data.

## Supporting Information Available

Bivariate polar plots for PM_2.5_ in the function of relative humidity and temperature on hourly raw, calibrated, and reference grade data sets as reported in this study (PDF)
