# Supplementary material for: Urban Air Quality Management at Low Cost Using Micro Air Sensors: A Case Study from Accra, Ghana
Source: ACS EST Air. 2024 Nov 6;2(2):201–14. doi: 10.1021/acsestair.4c00172 (PMC11833764; doi:10.1021/acsestair.4c00172)
Supplement: Supplementary file 1 — ea4c00172_si_001.pdf [file ea4c00172_si_001.pdf]

Urban air quality management at low-cost using micro air sensors: A case study from Accra, Ghana

Collins Gameli Hodoli[1,2,3*], Iq Mead[4], Frederic Coulon[5], Cesunica E. Ivey[6], Victoria Owusu Tawiah[7], Garima Raheja[8], James Nimo[9], Allison Hughes[9], Achim Haug[10], Anika Krause[10], Selina Amoah[11], Maxwell Sunu[11], John K. Nyante[11], Esi Nerquaye Tetteh[11], Véronique Riffault[12], Carl Malings[13]

[1]School of Environmental, Civil, Agricultural and Mechanical Engineering, College of Engineering, University of Georgia, Athens, GA 30602-6113, USA
[2]School of Sustainable Development, University of Environment and Sustainable Development, PMB, Somanya, Eastern Region, Ghana
[3]Clean Air One Atmosphere, Accra, Ghana
[4]MRC Centre for Environment and Health, Environmental Research Group, Imperial College London, W12 0BZ, UK5
[5]Cranfield University, School of Water, Energy and Environment, Cranfield, MK43 0AL, UK
[6]Civil and Environmental Engineering, University of California, Berkeley, Berkeley, CA, 94720-1234 USA
[7]Kwame Nkrumah University of Science and Technology, KNUST, Kumasi, Ghana
[8]Lamont Doherty Earth Observatory, Columbia University, Palisades, NY, 10027, USA
[9]Department of Physics, University of Ghana, Legon, Accra, Ghana
[10]AirGradient Ltd, Chiang Mai, 50180, Thailand
[11]Environmental Protection Agency, Accra, Ghana
[12]IMT Nord Europe, Institut Mines-Télécom, Univ. Lille, Centre for Energy and Environment, 59000 Lille, France
[13]Morgan State University, Baltimore, MD, 21251, USA & NASA Global Modelling and Assimilation Office, Goddard Space Flight Center, Greenbelt, MD, 20771, USA

[*] Corresponding author. Tel: 1-706-240-4356
E-*mail address*: collins.hodoli@uga.edu

CONTAINS

Figure S1: Bivariate polar plot at levels of relative humidity on hourly data for $PM_{2.5}$ using AirGradient raw (a) calibrated (b) and T640 (c) datasets.

Figure S2: Bivariate polar plot at levels of temperature on hourly data for $PM_{2.5}$ using AirGradient raw (a) calibrated (b) and T640 (c) datasets.

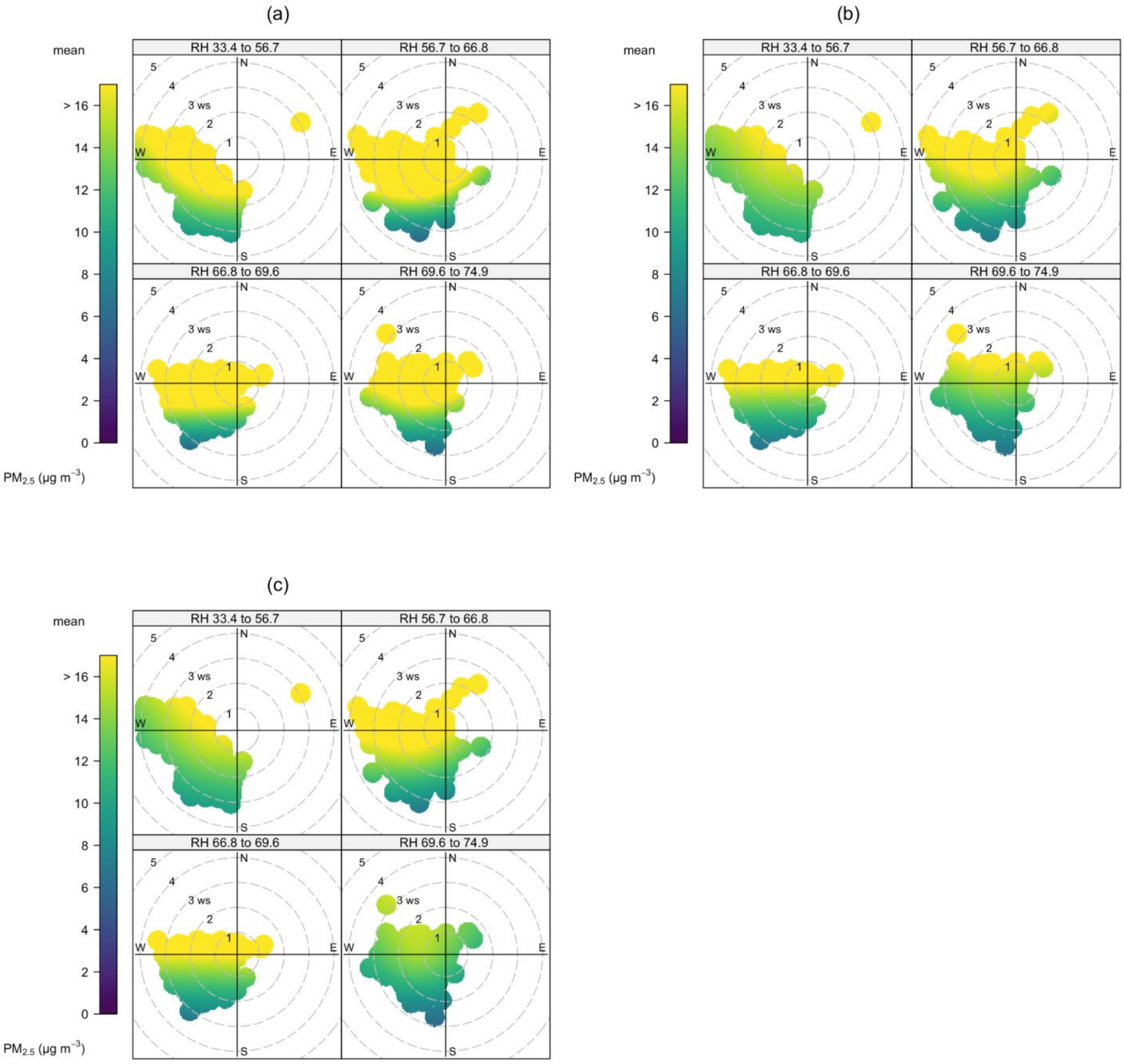

S 1: Bivariate polar plot at levels of relative humidity on hourly data for PM$_{2.5}$ using AirGradient raw (a) calibrated (b) and T640 (c) datasets.

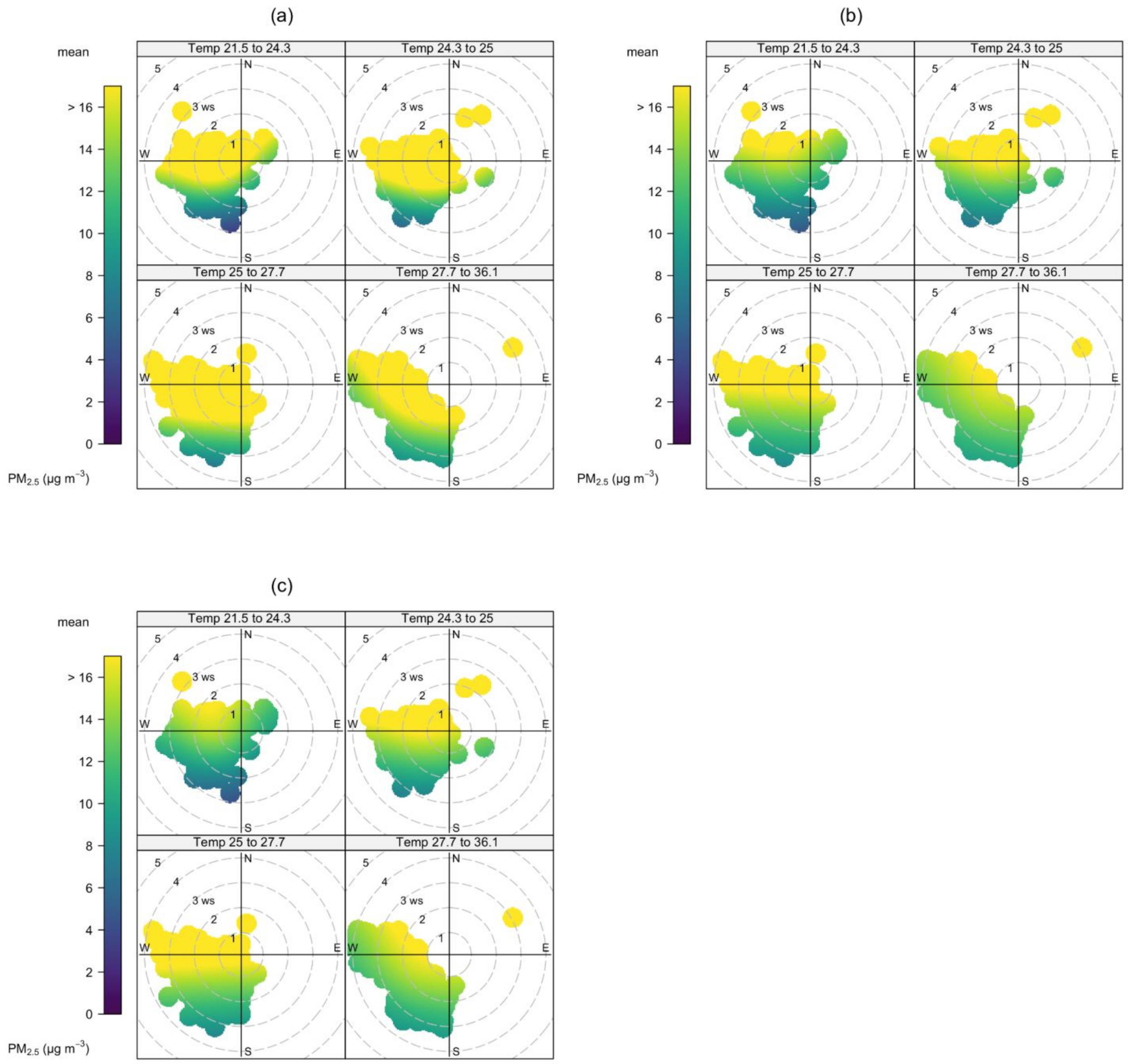

S 2: Bivariate polar plot at levels of temperature on hourly data for $PM_{2.5}$ using AirGradient raw (a) calibrated (b) and T640 (c) datasets.
